# Supplementary material for: Unscrambling fluorophore blinking for comprehensive cluster detection via photoactivated localization microscopy
Source: Nat Commun. 2020 Oct 5;11:4993. doi: 10.1038/s41467-020-18726-9 (PMC7536177; doi:10.1038/s41467-020-18726-9)
Supplement: Supplementary file 3 — Reporting Summary [file 41467_2020_18726_MOESM3_ESM.pdf]

nature research

Corresponding author(s): Mario Brameshuber and Johannes B. Huppa

Last updated by author(s): Sep 1, 2020

Reporting Summary

Nature Research wishes to improve the reproducibility of the work that we publish. This form provides structure for consistency and transparency in reporting. For further information on Nature Research policies, see our Editorial Policies and the Editorial Policy Checklist.

Statistics

For all statistical analyses, confirm that the following items are present in the figure legend, table legend, main text, or Methods section.

n/a	Confirmed	
☐	☒	The exact sample size (n) for each experimental group/condition, given as a discrete number and unit of measurement
☐	☒	A statement on whether measurements were taken from distinct samples or whether the same sample was measured repeatedly
☐	☒	The statistical test(s) used AND whether they are one- or two-sided<br>*Only common tests should be described solely by name; describe more complex techniques in the Methods section.*
☒	☐	A description of all covariates tested
☒	☐	A description of any assumptions or corrections, such as tests of normality and adjustment for multiple comparisons
☐	☒	A full description of the statistical parameters including central tendency (e.g. means) or other basic estimates (e.g. regression coefficient) AND variation (e.g. standard deviation) or associated estimates of uncertainty (e.g. confidence intervals)
☐	☒	For null hypothesis testing, the test statistic (e.g. F, t, r) with confidence intervals, effect sizes, degrees of freedom and P value noted<br>*Give P values as exact values whenever suitable.*
☒	☐	For Bayesian analysis, information on the choice of priors and Markov chain Monte Carlo settings
☒	☐	For hierarchical and complex designs, identification of the appropriate level for tests and full reporting of outcomes
☒	☐	Estimates of effect sizes (e.g. Cohen's d, Pearson's r), indicating how they were calculated

Our web collection on statistics for biologists contains articles on many of the points above.

Software and code

Policy information about availability of computer code

Data collection	Microscopy data collection was based on in-house developed code implemented in LabView2018 (Version 18.0f2) and software development kits provided by the camera manufacturer (ANDOR).
Data analysis	The ImageJ (2.1.0/1.53c) plug-in ThunderStorm (dev-2016-09-10-b1) was used to fit single molecule signals and construct SMLM localization maps. In-house developed code implemented in Matlab (R2019b) was used for channel registration, to analyze blinking behavior and for Ripley's K classification of experimental data by comparison with Monte Carlo simulated data. The complete software package for channel registration, the analysis of blinking behaviour and the comparison of cell-associated microscopy data with simulations is available for download via Github and Zenodo with the identifier(s) https://doi.org/10.5281/zenodo.4003734.

For manuscripts utilizing custom algorithms or software that are central to the research but not yet described in published literature, software must be made available to editors and reviewers. We strongly encourage code deposition in a community repository (e.g. GitHub). See the Nature Research guidelines for submitting code & software for further information.

Data

Policy information about availability of data

All manuscripts must include a data availability statement. This statement should provide the following information, where applicable:

- Accession codes, unique identifiers, or web links for publicly available datasets
- A list of figures that have associated raw data
- A description of any restrictions on data availability

Source data are provided with this paper. The data that support the findings of this study are available through "figshare.com" with the identifier(s) http://doi.org/10.6084/m9.figshare.1287153859. The datasets generated during and/or analyzed during the current study are available from the corresponding authors upon reasonable request.

Field-specific reporting

Please select the one below that is the best fit for your research. If you are not sure, read the appropriate sections before making your selection.

☒ Life sciences ☐ Behavioural & social sciences ☐ Ecological, evolutionary & environmental sciences

For a reference copy of the document with all sections, see nature.com/documents/nr-reporting-summary-flat.pdf

Life sciences study design

All studies must disclose on these points even when the disclosure is negative.

Sample size	Simulations were repeated 10-15 times. For each experiment on lipid bilayers or cells, sample size and the number of independent replicates are provided in the figure legends
Data exclusions	No data was excluded from the analysis.
Replication	Experiments were performed on n > 10 different positions on lipid bilayers as stated in the relevant figure legends. Results did not depend on the positions used for analysis. To obtain the final results all experiments/positions of a specific condition were pooled.
Randomization	This study did not include experiments for which randomization was applicable.
Blinding	Blinding was not relevant to this study because our experimental subjects are single molecule signals rather than patients.

Reporting for specific materials, systems and methods

We require information from authors about some types of materials, experimental systems and methods used in many studies. Here, indicate whether each material, system or method listed is relevant to your study. If you are not sure if a list item applies to your research, read the appropriate section before selecting a response.

Materials & experimental systems

n/a	Involved in the study	
☐	☒	Antibodies
☒	☐	Eukaryotic cell lines
☒	☐	Palaeontology and archaeology
☐	☒	Animals and other organisms
☐	☒	Human research participants
☒	☐	Clinical data
☒	☐	Dual use research of concern

Methods

n/a	Involved in the study	
☒	☐	ChIP-seq
☒	☐	Flow cytometry
☒	☐	MRI-based neuroimaging

Antibodies

Antibodies used	The CD11a (LFA-1alpha chain) reactive monoclonal antibody TS2/4 was purchased from BioLegend (Cat: 350602, Lot: B168865); the murine CD16/32 monoclonal antibody was purchased from BioLegend (clone 93, Cat: 101302); the CD28.2 reactive monoclonal antibody was purchased from eBioscience (Cat: 14-0289-82) and the human CD3epsilon reactive monoclonal antibody OKT-3 was purchased from eBioscience (Cat: 14-0037-82). The anti-mouse TCR beta chain reactive monoclonal antibody H57-597 was purchased from BioLegend (Cat: 109202).
Validation	Specific binding was verified by fluorescence microscopy (TS2/4 mAb and H57-597 mAb).<br>The TS2/4 monoclonal antibody was biotinylated as described in this manuscript and used to stain CD11a on human T cells; the H57-597 monoclonal antibody was biotinylated as described in this manuscript and used to stain the TCR beta chain of murine T cells. The murine CD16/32 monoclonal antibody was used for blocking of CD16/32 interactions with the Fc domain of IgGs (i.e. H57-597 mAb) as described by the manufacturer. The human CD28.2 reactive mAb and the human CD3epsilon monoclonal antibody were used for polyclonal human T cell stimulation. For further product details, specifications and application references please refer to the manufacturers declaration of the corresponding monoclonal antibody.

Animals and other organisms

Policy information about studies involving animals; ARRIVE guidelines recommended for reporting animal research

Laboratory animals	Primary T cells were isolated from 5c.c7 TCR transgenic B10.A male and female mice at an age of 8-12 weeks.
Wild animals	No wild animals were used in the study.

Field-collected samples	No field collected samples were used in the study.
Ethics oversight	Animal husbandry, breeding and sacrifice for T cell isolation were evaluated by the ethics committees of the Medical University of Vienna and approved by the Federal Ministry of Science, Research and Economy, BMWFW (BMWFW-66.009/0378-WF/V/3b/2016). All animal related procedures were performed in accordance with Austrian law (Federal Ministry for Science and Research, Vienna, Austria), the guidelines of the ethics committee of the Medical University of Vienna and the guidelines of the Federation of Laboratory Animal Science Associations (FELASA).

Note that full information on the approval of the study protocol must also be provided in the manuscript.

Human research participants

Policy information about studies involving human research participants

Population characteristics	Blood to isolate PBMC-derived T cells was drawn from adult female and male healthy donors younger than 53 years.
Recruitment	Healthy volunteers for blood donations were recruited among scientific colleagues active at the Medical University of Vienna.
Ethics oversight	The study using human material (PBMCs for the isolation of human T cells) was performed in accordance with the Declaration of Helsinki, informed consent was obtained from all participants and research was approved by the Ethics Committee of the Medical University of Vienna (2177/2013, 559/2005).

Note that full information on the approval of the study protocol must also be provided in the manuscript.
